# The implementation results of safe bicycle transport in Taipei City

**Published:** 2019-08

**Authors:** Wu Yin-Huan, Yang Ching-Ting, Chou Chen-Chen, Chang Man-Lin, Hsieh Ming-Yu, Kuo Yeh-Tzu

**Affiliations:** ^*a*^ Xinyi Safety and Health Association, Taipei City, Taiwan (Province of China).; ^*b*^ Xinyi District Health Center, Taipei City, Taiwan (Province of China).

## Abstract

**Background:**

In 2013, there were 7,016 bicycle accidents, resulting in 62 deaths and 10,162 injuries in Taiwan. Since then, there have been more than 7,000 accidents every year, and the number of accidents in 2017 has reached a peak of 8,106 records for the past five years. Among them, the number of electric bicycle accidents increased the most, with 1,183 compared to 2013 and the number of persons injured also increased by 1,310 whereas the number of bicycle accident from 6,262 to 6,169.

**Methods:**

Taipei City creating a friendly, safe and secure urban environment to ride:

1. Bicycle Road Network Deployment: bicycle-exclusive lanes, walkways, bicycle and pedestrian shared path and so on in order to meet a variety of traffic needs.

2. Safe and comprehensive road environment: Set up bike stands, road surfacing, street trees, signs, (pavement) markings, dedicated signs, intersection rotation and other traffic amenities to create a safe and comfortable riding environment. To promote cycling to the station to transfer MRTs is tantamount to encouraging people to take more public transport.

3. Planning a city bicycle rental system: To provide a public rental system to expand the convenience, visibility and the community of users launched a new type of public transport - public bicycle rental system (YouBike). The Youbike started demonstration operation, initially setting up 11 station points and 500 bicycles in the peripheral regions of Xinyi District on March 11th, 2009, and now there are 400 station points in all districts.

4. Bicycle Safe Riding Education: Most people refuse to wear helmet protection, and do not install safety devices such as reflectors and warning lights. Causes easy collision at night. The top three reasons for bicycle accidents are not giving way according to regulations, inappropriately making U-turns (turn direction) and Breaching traffic signs and sign controls. Furthermore, wrong-way driving and Careless overstepping are also the main causes of accidents.

5. Smart transportation Setting: To build a safe environment from the appropriate space. Its city area cycle path from 41 kilometers in 2006 (16.4 for dedicated lanes and 24.6 for bicycle and pedestrian shared path) to an extended 391 kilometers in 2018 (86.7 kilometers for dedicated lanes and 304.3 for bicycle and pedestrian shared path). The number of bike stands in Taipei urban area has increased from 5,202 in 2006 to 42,121 in 2018(809%). Appropriately adjust the lane allocation ratio through management means and let the left-turning bicycle turn in a two-stage left turn. This way allows the bicycle to start earlier than the steam locomotive when the green light starts to light up.

**Results:**

The implementation results of safe bicycle transport in Taipei City effectively reduced the death and rate caused by head injuries in bicycle accidents: the number of casualties (6,262 dropped to 6,169, down 1.49%), the number of deaths (59 to 30, a drop of 49.15%) or the number of injured persons (9,099 to 8,711), all presented a declining trend.

**Conclusions:**

Changing car-oriented road planning, complete bicycle infrastructure, and respecting the driving awareness of bicycle users is the best protection for the public. All policies should have comprehensive safety policies to ensure personal safety. Just like separate two types of transport, the overall design of the exclusive separation bicycle road, the policy of forcing all ages riding locomotives to wear helmets, etc. In the future, we will continue to plan for self-defense special road layout, intersection ramp configuration, traffic engineering and parking and transfer facilities, so that all roads in the city are safe and friendly, and we will become the first place for bicycle safety riding. Successful experience is extended to the whole country.

**Keywords:**

Bicycle safety, Road safety, Road traffic safety, Injury, Surveillance

